# Evaluation of Critical Flicker-Fusion Frequency Measurement Methods for the Investigation of Visual Temporal Resolution

**DOI:** 10.1038/s41598-017-15034-z

**Published:** 2017-11-15

**Authors:** Auria Eisen-Enosh, Nairouz Farah, Zvia Burgansky-Eliash, Uri Polat, Yossi Mandel

**Affiliations:** 1 School of Optometry and Vision Science, Faculty of life Sciences, Bar-Ilan University, Ramat-Gan, Israel; 2 E. Wolfson Medical Center, Holon, Israel; 3 Sackler School of Medicine, Tel-Aviv University, Tel-Aviv, Israel; 4 Bar Ilan’s Institute for Nanotechnology and Advanced Materials (BINA), Bar-Ilan University, Ramat Gan, Israel

## Abstract

Recent studies highlight the importance of the temporal domain in visual processing. Critical Flicker-Fusion Frequency (CFF), the frequency at which a flickering light is perceived as continuous, is widely used for evaluating visual temporal processing. However, substantial variability in the psychophysical paradigms, used for measuring CFF, leads to substantial variability in the reported results. Here, we report on a comprehensive comparison of CFF measurements through three different psychophysical paradigms: methods of limits; method of constant stimuli, and staircase method. Our results demonstrate that the CFF can be reliably measured with high repeatability by all three psychophysics methods. However, correlations (r = 0.92, p≪0.001) and agreement (Bland Altman test indicated 95% confidence limit variation of ±3.6 Hz), were highest between the staircase and the constant stimuli methods. The time required to complete the test was significantly longer for the constant stimuli method as compared to other methods (p < 0.001). Our results highlight the suitability of the adaptive paradigm for efficiently measuring temporal resolution in the visual system.

## Introduction

The human visual system processes visual information in two domains, namely: the spatial and temporal. Spatial resolution is defined as the ability to discriminate, in space, between two adjacent objects^1^ and is determined by several factors, such as the eye optics^2^, spatial organization of the photoreceptors cells^3^, the degree of neural convergence in the retina^4,5^ and higher visual areas in the brain. Temporal resolution is related to the temporal features of the stimuli and is defined as the ability to discern luminance changes over time^6^. The visual system’s temporal performance is limited by the finite time required for collecting and processing information^6^, and intermittent stimuli presented to the eye are perceived as separate only if the presentation rate is below a certain threshold^7–11^, defined as the critical flicker-fusion frequency (CFF).

Recent studies^12–14^ have demonstrated the importance of the temporal domain in visual processing and highlighted the complex space-time interplay. There are several methods for exploring temporal processing such as temporal masking^15,16^, Rapid Serial Visual presentations^12,17–19^ and CFF. It’s assumed that the CFF can reflect the basic temporal function of the visual system and is therefore a good measure of its performance. The CFF, is affected by a number of physical factors such as stimulus intensity, color, size, contrast and eccentricity, as well as light adaptation conditions and the subject’s age^20–24^. In addition to physical factors, the CFF can be reduced by various systemic medical conditions such as hepatic encephalopathy^25^ or eye diseases such as multiple sclerosis^26^, Age Related Macular Degeneration^27–30^ or cataract^31^.

Although the temporal resolution of the visual system can be studied by recording the electrophysiological response of the visual system (e.g. electroretinogram^32,33^ or visual evoked potentials to flickering stimuli^34,35^), in most settings it is usually evaluated through various psychophysical methods designed to measure the subject’s CFF value, as is described next. There are several major psychophysical paradigms^36–39^ widely used for measuring various sensory thresholds (e.g. vernier acuity^40^ and contrast sensitivity^41^ in vision, or auditory sensitivity^38^) with the widely used method of constant stimuli (MCS) being the most accurate of these methods^42^. This method is based on presenting the stimuli in a random order and therefore reduces errors caused by habituation and expectation. Moreover, it allows for the full sampling of the psychometric function^43^, which carries additional information. Notwithstanding, there are not many reports of this method being used for examining CFF in literature^44,45^ since it is time consuming and can be exhausting for the subject^43^. More commonly used, are the methods of limits (MOL)^19,25,46–53^ and the method of adjustment^30,54^, which are of significantly shorter duration and are therefore not exhausting to subjects. Nevertheless, these methods are considered less accurate as they are prone to subject bias^49,55^, which results from a set of internal rules governing the subject’s decision on whether or not a flicker is perceived at any given frequency^56–58^. Finally, the methods which are widely used for various psychophysical studies^41,55,59^ are variations of adaptive methods, such as the well-known staircase method (SM). The latter method combines high accuracy along with a short experimentation time as compared to the MCS^36,55^, and was therefore adopted by our group for measuring spatial functions^12,41,60–62^. Nevertheless, only a few studies reported the use of this method for measuring CFF thresholds (e.g.^31,63,64^).

Despite the relevance and applicability in clinical practice, there is lack of data regarding the agreement between the various paradigms used to measure CFF thresholds. In one such study, Feshchenko *et al*.^45^ compared the results obtained with MOL to those obtained by a short version of the MCS and found that both estimates of CFF thresholds differed by less than 0.1 Hz with the MCS providing higher reliability as compared with the MOL CFF estimation^45^.

In addition to differences in the psychophysical paradigm used for measuring CFF in various reports, there is also a lack of consistency with regards to the various stimuli parameters used for CFF measurements, such as stimuli light intensity, size and color^19,25,30,31,44,46–48,52–54,63–65^, thus adding more ambiguity to the results published in literature and making the comparison between the various methods difficult.

Here, we present a thorough comparison between the three psychophysical paradigms (MOL, MCS and SM) methods, in terms of CFF measurement repeatability, test duration and agreement. Moreover, we report on the effect of stimulus intensity and adaptation time on CFF thresholds. To perform these studies, we developed a robust and portable CFF measurement system using a LED (light-emitting Diode) controlled by a laptop, a data acquisition board, and a customized computer software. The system provides the basis for a reliable CFF measuring system which is inexpensive, highly portable and can be of high applicability in a clinical setting.

## Results

### The test apparatus

**Figure 1:**
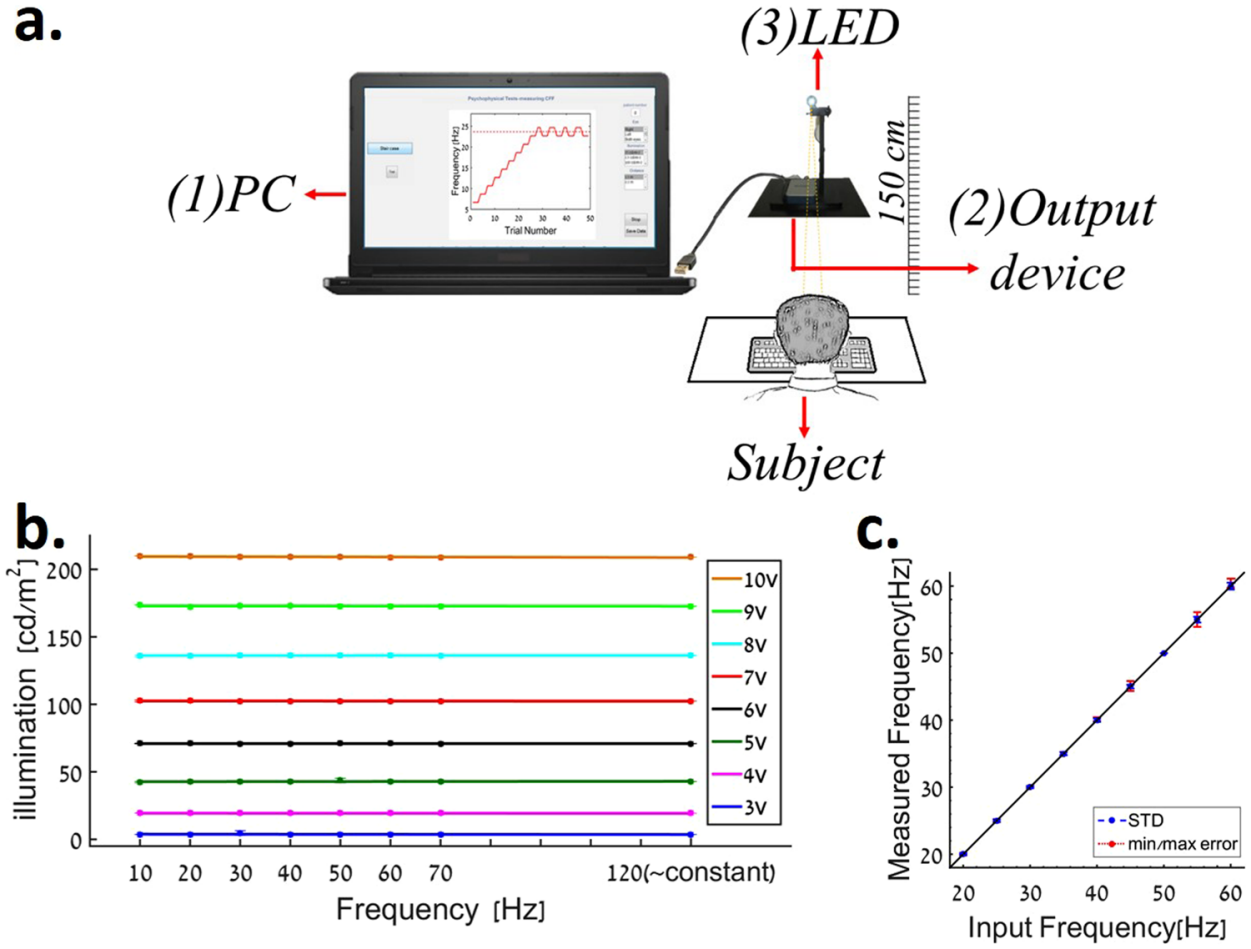
(**a**) Experimental setting. Subject seated 150 cm from a LED stimulus source, which is drived by a data acquisition board and a computer. (**b**) Luminance constancy. The average light intensity as a function of frequency (10Hz–70Hz and 120 Hz) was measured (6 repetitions) for various LED driving voltage. Bars represent standard error. (**c**) Frequency validation. Stimulus frequency, as measured by a recording system, plotted against the desired frequency.

We developed a CFF testing system based on a custom written Matlab code (The Mathworks, Waltham, Massachusetts) and an analog data output device, to drive a LED, control the flickering frequencies and analyze the perceived CFF threshold (Fig. 1a). We validated the precision of the stimulus frequency as described in the Methods section. More importantly, we excluded an illumination artifact by measuring a stable illumination along the entire range of the tested frequencies (Fig. 1b and Methods). The apparatus was placed at a distance of 150 cm from the subject, where the resulting stimulus size on the retina is 0.2 degree.

The stimulus flicker rate was varied between 10 to 60 Hz while a 120 Hz flickering light served as the constant stimulus (Fig. 1c and Methods). The Matlab software was further used to control the psychophysical test parameters (intensity, duration), as is described in the Methods section and Table 1.

**Table 1:** Description of three types of psychophysical tests.

	Starting point	Step size	Ending point	Threshold estimation
Method of limits	20 Hz for ascending 60 Hz for descending	2 Hz	Completion of a minimum of 3 iterations with a standard deviation smaller than 3 HZ	mean of all iterations
Method of Constant Stimuli	10 Hz below Method of limits-CFF	1 Hz	20 repeitions per each frequency, the end flickering frequency is 10 Hz above Method of limits CFF	Create sigmoid curve from the results and determination of CFF in 80%
Staircase method 3 up/1 down	18 Hz below Method of limits-CFF	2 Hz	Completion of 8 reversals	mean of the last 6 reversals

All participants had no strabismus, exhibiting normal visual acuity and stereopsis with no ocular or neurological disease. All experiments were performed on each eye separately with both eyes opened while occluding the non-tested eye by an opaque lens mounted on glasses frame. The experiments were assessed in a dark room similar to previous reports^19,52^.

### CFF measurements showed high test-retest repeatability

**Figure 2:**
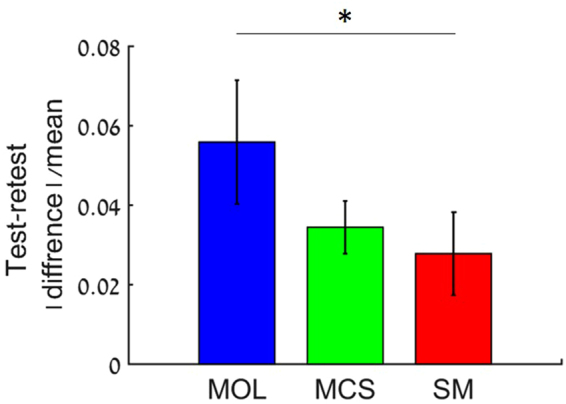
Test-retest of CFF obtained by the three methods. CFF values were measured at illumination levels of 40 and 100 cd/m^2^. Test-retest CFF difference over the mean is presented for the three methods (n = 10). Mean test-retest(SE) 0.06 (0.015), 0.03 (0.006) and 0.028 (0.01) for MOL, MCS and SM, respectively. *p = 0.05.

In order to evaluate the repeatability of the methods, the CFF threshold was measured using each methods twice on different days at stimuli luminance levels of 100 cd/m^2^ and 40 cd/m^2^. Test-retest repeatability of the three methods was then estimated using the formulation: test-retest = $\frac{| {\rm{diffrence}}| }{{\rm{mean}}}$, where difference is the absolute difference between two paired measurements and the mean is the mean of the two measurements (Fig. 2). Although all three methods showed high repeatability, both the SM and the MCS showed significantly higher repeatability with smaller test-retest difference as compared with the MOL (p = 0.05). As an additional measure of repeatability, we analyzed the correlation between the results of the first test with the results of the re-test for each method^66^. The highest correlations were found for SM (r = 0.95, p ≪ 0.001) and MCS (r = 0.946, p ≪ 0.001) while MOL showed lower correlation (r = 0.72, p ≪ 0.05), highlighting the lower repeatability of this method (data not shown).

### Correlation and agreement between the various methods

**Figure 3:**
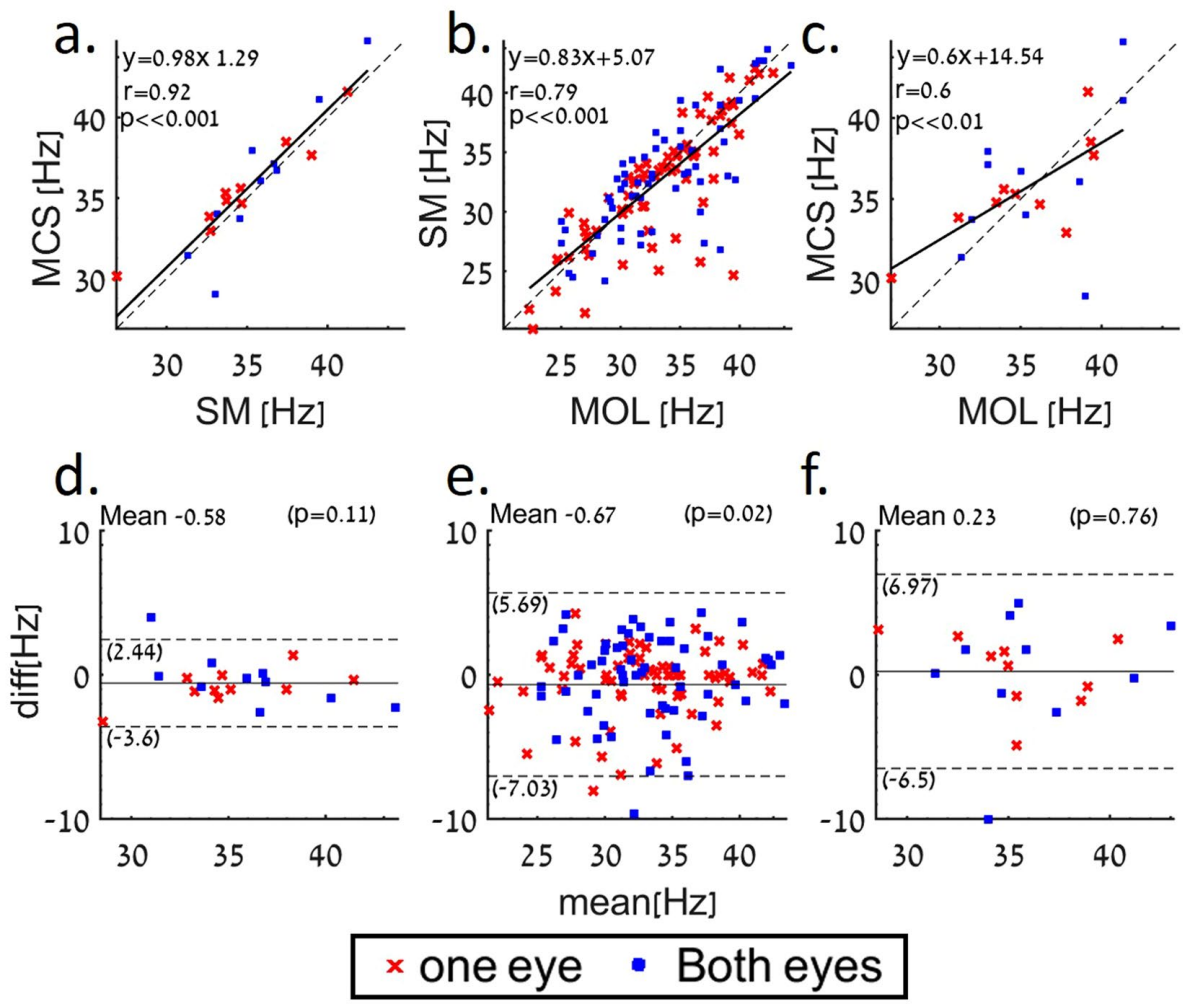
Correlations and agreement between the various methods. (**a,b,c**) The correlations between CFF values obtained by the three methods are presented for all stimuli luminance for 10 subjects (6 females, 4 males). (**a**) The CFF correlation between SM and the MCS. (**b**) The CFF correlation between the MOL and the SM. (**c**) The CFF correlation between the MOL and the MCS. (**d**–**f**) Bland–Altman plots of CFF values. Dashed lines-mean ± 1.96sd.d) A Bland–Altman plot of SM and MCS. (**e**) A Bland–Altman plot of MOL and SM. (**f**) A Bland–Altman plot of MOL and MCS.

To evaluate the correlation and agreement between the three methods, the CFF threshold was measured at six different luminance levels in the range of 2.5–100 cd/m^2^ using MOL and SM, and at a luminance level of 40 cd/m^2^ using the MCS. We found a high and statistically significant correlation between the MCS and the SM tests (r = 0.92, p ≪ 0.001. Figure 3a). Moderate correlations were found between the other pairs of tests (Fig. 2b,c). We further analyzed the agreement between the different CFF tests through Bland–Altman tests^67,68^. Again, the highest agreement was found between the SM and MCS, with a maximum difference of 3.6 Hz (Fig. 3d), while other comparisons showed a moderate smaller agreement (Fig. 3e,f).

It should be noted that for the same luminance levels, we observed low variability between subjects (SE 0.9–1.7), in agreement with previous results in young healthy subjects^46,48,53^.

### Test duration Effect

**Figure 4:**
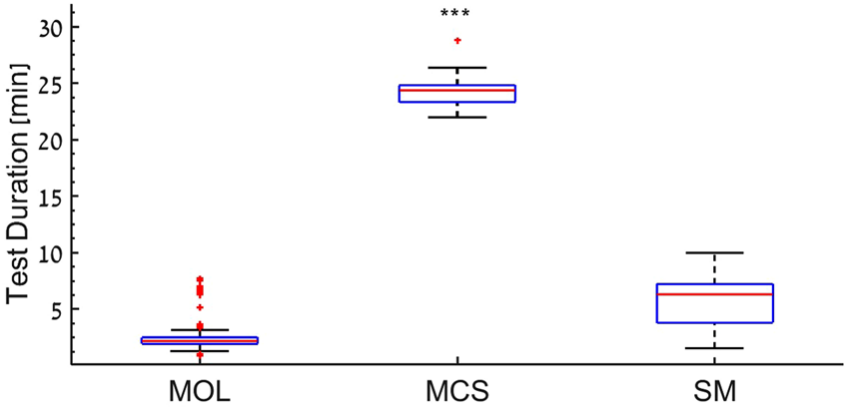
Test duration. Durations of each method for CFF measurement are plotted in minutes. The duration of the MCS test (median 24.33 min, maximum 41 min, minimum 22 min) is significantly longer than that of the two other tests (p < = 0.001). The durations of the SM test was median 6.3 min (maximum 9 .96 min, and minimum 1.95 min) and the durations of MOL test was median 2.2 min (maximum 7.73 min, minimum 0.935 min) ***p < = 0.001.

To avoid subject fatigue which may introduce bias and reduce reliability, it is important to minimize test duration. Therefore, we measured the test duration of the three paradigms (Fig. 4). The median test time of the MCS test (24.33 min) was found to be significantly longer than the other two paradigms: 3 times longer than SM (6.3 min, p < 0.00), and 11 times longer than MOL (2.2 min, p < 0.001). The SM test duration was approximately 4 mins longer, as compared with the MOL (p < 0.001). We further evaluated the effect of subject fatigue along the MCS test sessions by analyzing the change in CFF values obtained along the 20 iterations comprising each test session. We found that the average CFF threshold of the first 10 iterations in the MCS method was 0.8 Hz higher (range 1.1–3.4 Hz difference) as compared to the average CFF threshold of the last 10 iterations (p = 0.049) (Figure S2a). This small but statistically significant difference could be the effect of fatigue and can lead to an underestimation of the CFF threshold when measured with the MCS. In contrast, in the SM data, our analysis showed low correlation between number of iterations and final CFF threshold (r = 0.25), suggesting little or no fatigue effect in the SM method (See Figure S2b).

### Light adaptation time effect of on the CFF

**Figure 5:**
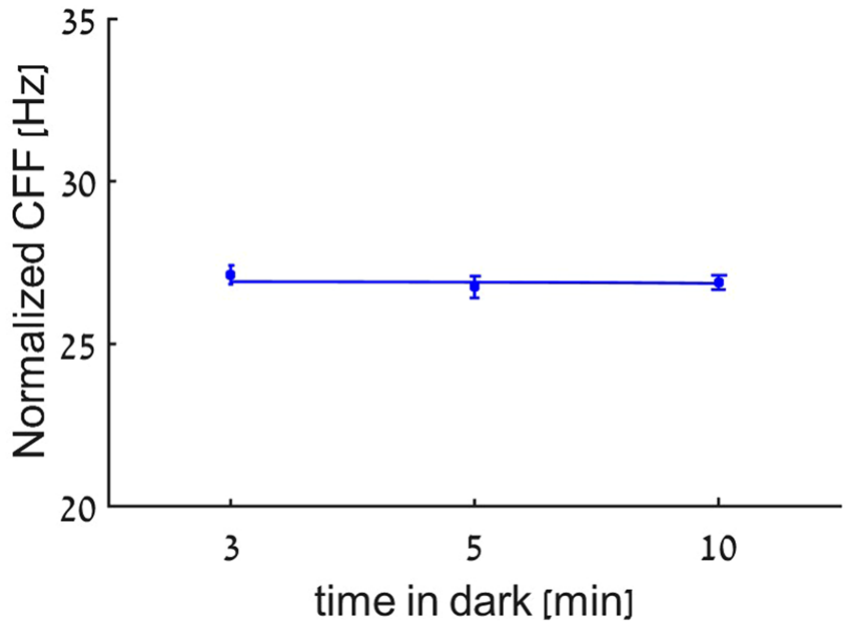
Dark adaption effect on CFF. Mean CFF values as a function of dark adaptation time is plotted for 10 subjects.

In order to study the effect of light adaption on the CFF threshold, we measured CFF thresholds at various time-points of dark adaptation. Our results revealed that a steady CFF threshold is achieved after 3 minutes of dark adaptation with no significant difference observed between the measured values for 3, 5 and 10 minutes of dark adaptation (Figure 5) (P = 0.55, P = 0.45, P = 0.79, respectively, indicating that a dark adaptation time of 3 minutes prior to each measurement, is sufficient.

### Effect of stimulus luminance

**Figure 6:**
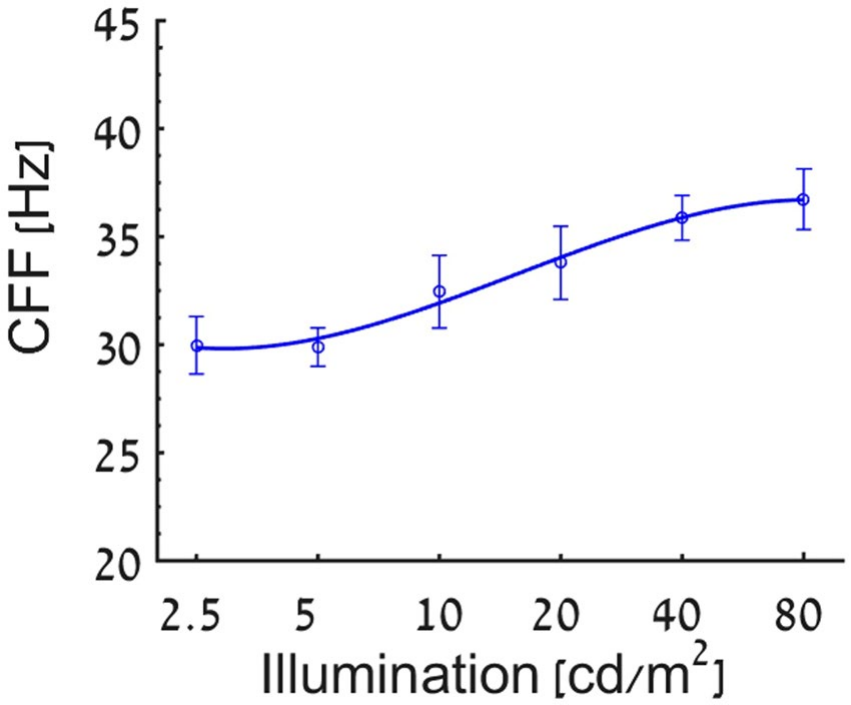
The Effect of Luminance: CFF values are presented as a function of stimuli luminance levels for 10 subjects.

In order to evaluate the effect of stimulus luminance level on the temporal resolution, CFF tests were performed at six different luminance levels ranging between 2.5–80 cd/m^2^ using the SM. Increasing stimulus luminance yielded higher CFF values, reaching a plateau around 80 cd/m^2^ (Fig. 6) with a mean difference of 6.7 Hz (~20%) between the higher to lower luminance levels.

## Discussion

In this study, we present a comprehensive comparison between three methods for measuring CFF threshold in the aim to single out the most time efficient, reliable and repeatable method. Towards this end, we developed a measuring system, based on a laptop, which controls a LED (See Figs 1, S1) and enables an easy measurement of CFF thresholds in three methods, MOL, SM and MCS.

Our results demonstrate the high repeatability of the various CFF test paradigms with small variability between test repetitions in all three methods. Repeatability, however was better for the MCS and SM tests as compared with the MOL test (Fig. 2). High correlation and good agreement was observed between the various paradigms used in this study. More importantly, the highest agreement and correlation values were observed between the SM and the MCS tests, which is considered the gold standard in many psychophysical studies (Fig. 3a,d). In addition, the SM was found to be significantly shorter as compared to the MCS (Fig. 4), making it more advantageous in both research and clinical settings.

To the best of our knowledge, this is the first comprehensive comparison between the various psychophysical methods for the evaluation of temporal resolution. To date, the MOL is widely used for measuring CFF^19,25,46,47,49^ thresholds, albeit its known subject response bias^69,70^. The MCS test, although considered the most accurate in the psychophysical field^42^ for measuring various sensory thresholds, has not been widely used for the evaluation of CFF thresholds, with only a few studies reporting on using it (e.g Carmel *et al*.^44^). The long test duration makes the use of the MCS method for evaluating the CFF threshold in a clinical setting difficult, mainly when the investigation of the effect of various test conditions is of interest (e.g. stimuli illumination). More importantly, the long test duration also introduces an effect of fatigue or loss of attention, with up to 3.4 Hz effect, as was found in our study (Figure S2a) as well as by previous reports^55,71^.

The good agreement between the SM and MCS (Fig. 3) tests, the short test duration (Fig. 4) and small test-retest variability (Fig. 2), make the SM the method of choice and a good alternative to the MCS for measuring CFF thresholds, similar to the wide use of the SM for measuring spatial resolution^55,71^.

An important factor which was found to affect CFF thresholds is the ambient light conditions which modulate both the spatial and temporal sensitivity of the visual system^20^. Although it is commonly agreed on that cone photoreceptors adaptation takes about 5–10 minutes and rod photoreceptors adaptation can take up to 30 minutes^72^, our results demonstrate that the CFF threshold reached a plateau level in a dark room setting after a short 3 minutes adaptation period (Fig. 5). It should be noted that evidence of such fast dark adaption for spatial performance measurements were reported, when stimuli were presented to the fovea^73^, similarly to the current study (the stimulus size was 0.2 degree).

The optimal method for measuring CFF threshold should be accurate and reproducible in order to enable detection of small changes in CFF values over time. Furthermore, since the test is usually conducted as a part of a long battery of other psychophysical tests, it should be of short duration in order to avoid subject exhaustion which can introduce bias to the results^74^. The described staircase paradigm was found to be of short time and an accurate alternative to the longer tests and can therefore be efficiently used in research and clinical settings. Future studies may further explore the suitability of other adaptive methods (e.g.^75–77^) for the measurement of CFF threshold.

In conclusion, we presented a comprehensive comparison between three psychophysical methods used for the evaluation of CFF thresholds. A simple laptop based setup can be used to test subjects and patients with a short and highly repeatable CFF measurements based on a staircase paradigm and aid in the evaluation of temporal processing in the visual system.

## Methods

### Apparatus

We developed a novel laptop based CFF measuring system based on a custom written Matlab code (The Mathworks, Waltham, Massachusetts) and an analog data output device (sampling rate 5000 Hz, National Instruments NI-USB-6001), to drive an LED, control the flickering frequencies and analyze the perceived CFF threshold (Figure S1). Using a sampling rate of 5,000 Hz the device produced a sine wave to drive the LED stimuli with 100 percent modulation depth. We used a Cool White ‘Cree® 5 mm round LED^78^ with a diameter of 5 mm and controlled the stimuli light intensity and frequency by setting the amplitude and frequency of the output derived by one of the available analog outputs on the board. Frequency validation was performed by recording the generated electrical signal using an electrophysiological recording system (AlphaLab SnR^TM^, Alpha Omega LTD, Israel) over 1 second. Results revealed (Fig. 1c **)** that the measured frequency is stable and with a very small variation from the intended frequency (STD of maximum ±0.5 Hz with a maximum error of 1.2 Hz observed only at the higher end of the employed frequencies (55–60 Hz).

We calibrated the required voltage needed to elicit a desired LED illumination level (measured using a photometer -‘Konica Minolta LS-110) at a range of up to 100 cd/m². A neutral density filter of 1OD was used to display the lower light intensity range of 2.5–5 cd/m². Note that in order to avoid luminance artifacts, caused by luminance differences between various stimuli frequencies, luminance levels were carefully measured over the entire range of tested frequencies demonstrating a constant illumination level with a maximal difference of 0.256 cd/m^2^ in the frequency range of 10–120 Hz. (Fig. 1b). Experiments were performed to ensure that these differences are indeed undetectable with results validating that the employed illumination levels were perceived as constant (Figure S3). Stable illumination of more than 200 cd/m^2^ could be produced by the device, however in the current study we used a maximal illumination of 100 cd/m^2^.

### Participants

The various CFF tests were performed on ten healthy participants (6 females, 4 males age 27.15 ± 2.91 years old, mean ±STD) with no known neurological conditions and with normal corrected vision. The study was conducted according to the guidelines and regulations for human subject research. All participants signed an informed consent form and the study was approved by and conducted according to both the IRB Committee at the Edith Wolfson Medical Center, Holon, Israel (Application number 0108-15-WOMC, Holon, Israel) and by the Bar-Ilan University Ethics Committee guidelines.

All participants underwent a comprehensive vision examination by a qualified optometrist (A.E) including far and near visual acuity (ETDRS charts), with full optical correction. All participants were refracted by dry retinoscopy, and tested for binocular ‘Randot stereo’ test, cover-test and underwent general eye examination including fundus ophthalmoscopy and a slit lamp examination of the anterior segment. The criteria for inclusion were visual acuity better than 0.1 LogMar with a difference of less than 0.2 LogMar between eyes, stereopsis better than 40″ and no ocular or neurological disease. The mean stereopsis of the subjects group was better than 40″, mean visual acuity (logMAR) was: far monocular: −0.06, far binocular −0.14; near monocular: 0, near binocular −0.03.

### CFF test paradigms

**Figure 7:**
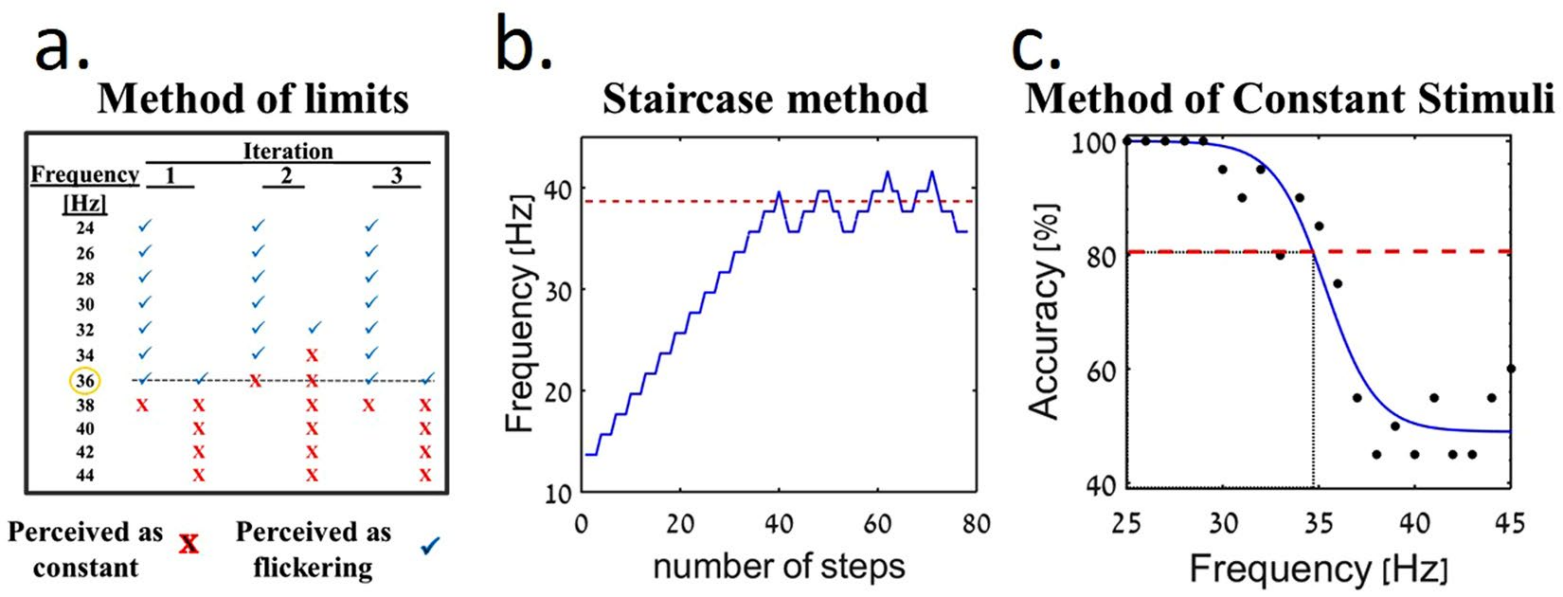
Schematic representation of the three tests. (**a**) Method of limits CFF = 36 Hz, (**b**) Method of Constant Stimuli CFF = 34.7 Hz (**c**) Staircase method CFF = 38.7 Hz.

Experiments were designed to evaluate the CFF threshold through three well known psychophysical tests, based on a discrimination task with a stimulus duration of 1 sec as described in Table 1 and Fig. 7. In the first, the MOL, stimuli with increasing (starting at 10 Hz) or decreasing (starting at 60 Hz) flickering frequency were presented to the subject until the subject reported on perceiving the stimulus as constant or flickering, respectively. The threshold calculated for a single trial was calculated as the average of three repeated iterations^55,56^. In order to reduce test variability, the trial was repeated until the CFF standard deviation of three consecutive iterations was smaller than 3 Hz and the threshold was then calculated as the mean of these three iterations. If this criterion was not met, then the trial was completed after 9 iterations and the threshold was calculated as the mean of all 9 iterations.

The other two tests (MCS and SM) were implemented using the two temporal alternative forced-choice paradigm (2TAFC), commonly used to eliminate the effect of response bias^37,69,79^. In these two tests, participants had to discriminate between a target stimulus, flickering light at the various frequencies and a flickering light at a high frequency of 120 Hz (significantly higher than the CFF in humans, therefore perceived as constant light). A high frequency, rather than constant light, was used in order to avoid luminance artifact.

In MCS, stimuli flickering at various frequencies were presented to the participants, in a random order, in a two-alternative forced-choice paradigm. Each frequency condition was repeated 20 times^36,44^ and the percentage of correct answers was then calculated. The threshold is determined after fitting the results into a logistic function^55,74^ and obtaining the well-known psychometric curve and then defining the CFF threshold at 80% correct level^80^. Note, that When comparing fitting quality obtained using the logistic function to one of the other available analysis functions (Weibull), with the mean square error as a measure, it was found to produce a better fit (mean square error of 0.2 as compared to 0.1, data not shown).

The SM was performed by modifying the stimulus frequency according to the participant’s response. We used an adaptive 3:1 method wherein the stimulus frequency is increased by one step in case of three consecutive correct responses and is decreased by one step in case of an incorrect response^81^. The frequency step size was 2 Hz. Under these conditions, the probability that the participant’s response is by chance or due to attention lapses under the assumption that these are independent events, is given by: $p(i=3)={0.5}^{3}=0.125$. The test was finalized upon the completion of 8 reversals (change in direction of the stimulus frequency). The CFF threshold was then defined as the mean of the last 6 reversal values, yielding a CFF calculation at a 79% correct level^13^^,^^41,59^. The entire procedure was repeated twice and the final CFF was calculated as the mean of the two repetitions. In order to further reduce test time, the staircase test started at a flickering frequency value of 18 Hz lower than the CFF measured using the MOL test.

As previously mentioned^20,72^ dark adaptation and ambient light conditions can have a significant effect on CFF. To reduce effects of lighting conditions on the experimental results all experiments were performed under dark room conditions, similar to previous reports^19,52^. Furthermore, in order to evaluate the effect of dark adaptation^72^^,^^82^ time on the CFF, we measured CFF thresholds following 5 min adaptation to a well-lit room (ambient light of 54.4 cd/m^2^) after which measurements were performed following 3, 5 or 10 minutes of dark adaptation (ambient light of 0.001 cd/m^2^). These tests were performed in a mixed order and with a 2.5 cd/m² flickering stimulus using the SM, as described above.

## Electronic supplementary material

Supplementary Information
