# Supplementary material for: Evaluation of Critical Flicker-Fusion Frequency Measurement Methods for the Investigation of Visual Temporal Resolution
Source: Sci Rep. 2017 Nov 15;7:15621. doi: 10.1038/s41598-017-15034-z (PMC5688103; doi:10.1038/s41598-017-15034-z)
Supplement: Supplementary file 1 — Supplementary Information [file 41598_2017_15034_MOESM1_ESM.pdf]

Evaluation of Critical Flicker-Fusion Frequency Measurement Methods for the Investigation of Visual Temporal Resolution

Auria Eisen-Enosh (1), Nairouz Farah (1), Zvia Burgansky-Eliash (2,3), Uri Polat (1), Yossi Mandel (1)*

(1) School of Optometry and Vision Science, Faculty of life Sciences, Bar-Ilan University, Ramat-Gan, Israel,

(2) E. Wolfson Medical Center, Holon, Israel

(3) Sackler School of Medicine, Tel-Aviv University, Tel-Aviv, Israel

* yossi.mandel@gmail.com

Supplementary

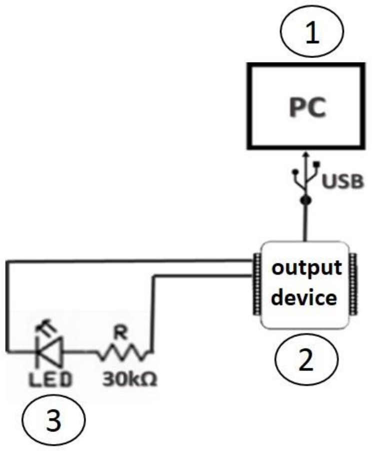

Figure S1: Device circuitry: (1) PC equipped with a Matlab software was used to drive a 'National Instruments' NI-USB-6001 (2). The stimulus was generated using (3) a Cool White 'Cree® 5mm Round LED'.

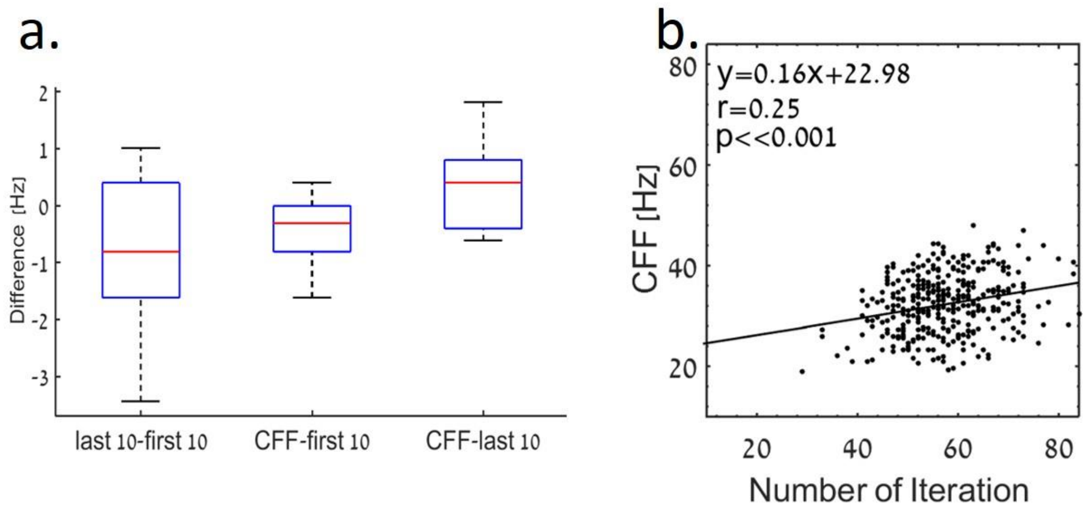

Figure S2: The effect of fatigue on CFF. (a) The difference between mean CFF values, first and last ten iterations as measured by MCS. (b) The correlations between CFF values to the number of iterations needed to evaluate CFF values in the SM.

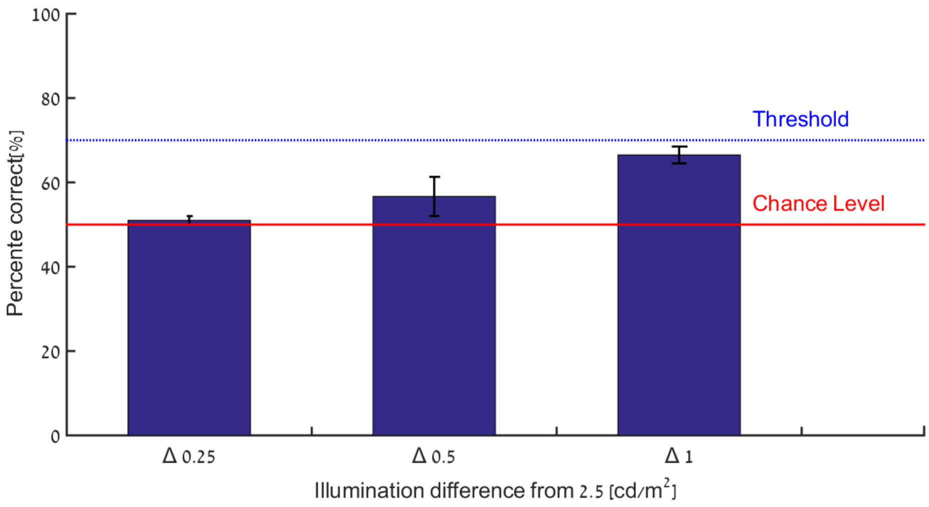

Figure S3: Detection of brightness difference. Five subjects (2 females, 3 males) were asked to determine the brighter light in a two-alternative forced choice paradigm. Baseline stimulus illumination level was 2.5 cd/m2 with luminance difference set at 0.25, 0.5 or 1 cd/m2. For a luminance difference of 0.25 cd/m2 the detection was 49.8%, SE=1, not different from chance level (p=0.86). However, when the same experiment was performed for a difference of 1 cd/m2 the detection level was ~70% correct. The results suggest that luminance artifact did not significantly affect our results.
